# Brittle Bone Disease: A Case Report

**DOI:** 10.7759/cureus.31259

**Published:** 2022-11-08

**Authors:** Tatiana Luis, Ana Cristina Gonçalves, Eduardo Rodrigues, Maricela Mendes, Tânia Teixeira

**Affiliations:** 1 Family Medicine, Serviço de Saúde da Região Autónoma da Madeira, Entidade Pública Empresarial da Região Autónoma da Madeira, Funchal, PRT

**Keywords:** family medicine, genetics, fractures, newborn, osteogenesis imperfecta

## Abstract

Osteogenesis imperfecta (OI) is a rare genetic disorder. Due to considerable phenotypic variability, a classification was developed for OI subtypes based on clinical features and disease severity.

A seven-day-old female was born at 40+1 weeks of gestation whose mother received routine antenatal care and had an uneventful pregnancy. In delivery, the newborn suffered bilateral collarbone fractures. After a week, she returned to an unscheduled appointment at the healthcare family unit due to an inconsolable cry and pain during mobilization of the left lower limb with three days of evolution, which were noticed by her parents. On examination, she presented edema in the right coxofemoral joint, asymmetry in the folds, and inconsolable crying during the mobilization of both hip joints. She was sent to the emergency department, where a pelvis X-ray was performed revealing a bilateral fracture of the femurs. During hospitalization, a genetic study revealed pathogenic variants of the *WNT1* gene, which causes OI type XV.

When a newborn presents with fractures, the main differential diagnosis is physical abuse. However, this was ruled out as we knew her mother and family, leaving no other possible evidence of abuse. OI was a highly probable diagnostic hypothesis due to the presence of two other cases of this type of OI in the same region of origin, even though her parents were not consanguineous and there was no history of fractures in their families.

Although OI is a rare condition, the diagnosis was immediately suspected because there were two confirmed cases of this type in the same geographic area as our patient. Additionally, she had bilateral clavicle fractures at birth with no obvious signs or risk factors for abuse. As family doctors, it is our aim to support this family throughout their journey and provide the child with the best care possible.

## Introduction

Osteogenesis imperfecta (OI) or brittle bone disease is a rare genetic disorder occurring in 1 in 15,000 to 20,000 births and is characterized by bone fragility and osteopenia [1]. Due to considerable phenotypic variability, a classification of OI types was developed based on clinical features and disease severity. Most forms of OI are autosomal dominant with mutations in one of the two genes that code for type I collagen alpha chains, namely, *COL1A1* (120150) and *COL1A2 *(120160) [2]. OI type XV is described as an autosomal recessive form of the disorder characterized by early onset of recurrent fractures, bone deformity, significant reduction of bone density, short stature, and, in some patients, blue sclera. Tooth development and hearing remain normal. Learning and developmental delays and brain anomalies have been observed in some patients [3,4].

## Case presentation

**Figure 1:**
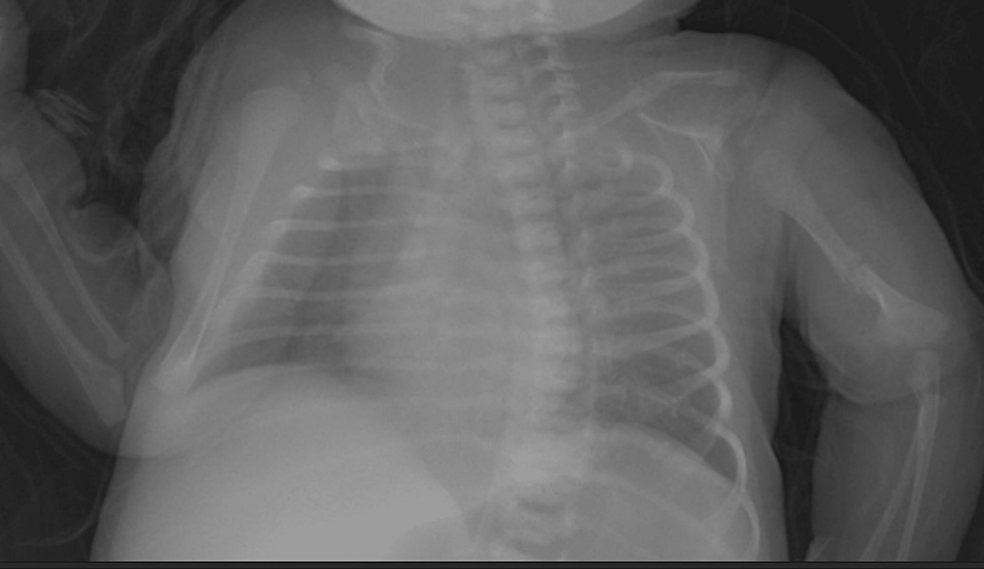
Radiography showing bilateral clavicle fracture and left humerus fracture

**Figure 2:**
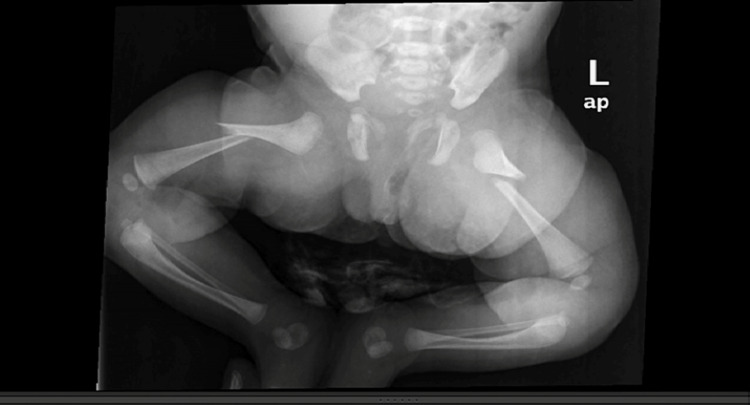
Radiography from the emergency room showing a bilateral shaft of the femur fracture.

A seven-day-old female was born at 40+1 weeks of gestation whose mother received routine antenatal care along with an uneventful pregnancy. Prenatal ultrasonography detected no fractures or other abnormalities. The baby was born in eutocic delivery without complications, with an Apgar score of 9/10 and the following anthropometric data: weight 3,630 g, length 47.50 cm, and cephalic perimeter 32 cm. At birth, she had a bilateral clavicle fracture (Figure 1) with left brachial paresis. Within a week of the life of the baby, she returned to an unscheduled appointment at the healthcare family unit due to an inconsolable cry and pain during mobilization of the left lower limb within three days of evolution, which was noticed by her mother. On observation, there was edema in the right hip joint, asymmetry in the folds, and inconsolable crying during the mobilization of both hip joints. She was sent to the emergency department, where a pelvis X-ray was performed revealing a bilateral diaphyseal fracture of the femurs (Figure 2), requiring hospitalization with the use of plaster for six weeks. During hospitalization, a genetic study was performed for OI (whole exome sequencing-based next-generation sequencing panel of 26 genes, including copy number variation analysis) revealing the probable pathogenic variant c.324 dup p.(Gly109Argfs*46) in probable homozygosity in the *WNT1* gene. This variant is not described in the literature nor the ClinVar database and is present in the gnomAD population database (only one heterozygous individual was reported). Pathogenic variants in the *WNT1* gene cause, among other pathologies, OI type XV, with an autosomal recessive inheritance. Thus, this result confirms a genetic etiology for the clinical picture presented. A genetic study was recommended and performed on the parents to confirm homozygosity and establish their carrier status. Currently, we are waiting for the results.

## Discussion

OI is known to be a collagen-related disorder caused by defects in collagen structure as well as in genes that affect collagen folding, post-translational modification and processing, bone mineralization, and osteoblast differentiation [5].

OI type XV is the result of homozygous mutations in *WNT1*. Heterozygous mutations in *WNT1 *cause dominantly inherited early-onset osteoporosis, being the genetic test of parents fundamental to diagnosing and treating this condition as soon as possible [5].

Even though collarbone fractures may occur in newborn babies, bilateral clavicle fractures are extremely rare [6]. Because the newborn was neither macrosomic nor post-term and did not have an instrumented delivery, the bilateral clavicle fracture was not expected. When a newborn presents with fractures, the main differential diagnosis is physical abuse or fall [7]. This was ruled out after confirming with the mother and other family members. Moreover, there was no other evidence of abuse, such as skin lesions and the fractures did not present specific patterns. OI was a highly probable diagnostic hypothesis due to the existence of two other cases of OI in the same region of origin, although her parents were not consanguineous and there was no history of similar fractures in both parents’ families. Considering the possible brain abnormalities, learning, and/or developmental delay associated with OI XV, these patients should be followed up by a multidisciplinary team and promote early involvement and family support [8,9].

## Conclusions

Although OI is a rare condition, the diagnosis was immediately suspected after assessment in the healthcare family unit because there were two confirmed cases of a similar condition in the same geographic area as our patient’s. Additionally, she had bilateral clavicle fractures at birth with no obvious signs or risk factors of abuse. The suspicion of abuse should always be the first thing to rule out in newborns with multiple fractures. The diagnosis can only be verified by relevant genetic testing, and even though there is no apparent relation between the other cases, there might be a role of shared family history in the past. As family doctors, we are obliged to support this family throughout their journey providing the best possible care for the child.
